# Modeling cardiac β-adrenergic signaling with normalized-Hill differential equations: comparison with a biochemical model

**DOI:** 10.1186/1752-0509-4-157

**Published:** 2010-11-18

**Authors:** Matthew J Kraeutler, Anthony R Soltis, Jeffrey J Saucerman

**Affiliations:** 1 Department of Biomedical Engineering, University of Virginia, Charlottesville, VA 22908, USA

## Abstract

**Background:**

New approaches are needed for large-scale predictive modeling of cellular signaling networks. While mass action and enzyme kinetic approaches require extensive biochemical data, current logic-based approaches are used primarily for qualitative predictions and have lacked direct quantitative comparison with biochemical models.

**Results:**

We developed a logic-based differential equation modeling approach for cell signaling networks based on normalized Hill activation/inhibition functions controlled by logical AND and OR operators to characterize signaling crosstalk. Using this approach, we modeled the cardiac β_1_-adrenergic signaling network, including 36 reactions and 25 species. Direct comparison of this model to an extensively characterized and validated biochemical model of the same network revealed that the new model gave reasonably accurate predictions of key network properties, even with default parameters. Normalized Hill functions improved quantitative predictions of global functional relationships compared with prior logic-based approaches. Comprehensive sensitivity analysis revealed the significant role of PKA negative feedback on upstream signaling and the importance of phosphodiesterases as key negative regulators of the network. The model was then extended to incorporate recently identified protein interaction data involving integrin-mediated mechanotransduction.

**Conclusions:**

The normalized-Hill differential equation modeling approach allows quantitative prediction of network functional relationships and dynamics, even in systems with limited biochemical data.

## Background

The β-adrenergic signaling pathway plays a key role in the regulation of normal heart function and the development of heart failure [1-5]. Systems analysis of β-adrenergic signaling in the heart may provide important new insights into the mechanisms of heart failure and reveal new therapeutic targets. Previous mathematical models of cardiac β-adrenergic signaling have characterized how biochemical mechanisms of this pathway determine its coordinated regulation of cell contractility in health and disease [6-8]. However, this work relied on extensive biochemical data from the literature that may not be available for more recently discovered pathways. Therefore, more scalable modeling approaches are needed.

As an alternative to generating biochemically detailed kinetic models, several modeling approaches that are more closely based on network topology have been developed including Boolean modeling [9], fuzzy logic modeling [10] and extreme pathways analysis [11]. These approaches require few or no parameters and facilitate large-scale analysis of systems properties, such as feedback loops and feasible solution spaces. But these approaches have a variety of limitations. While extreme pathways analysis predicts the entire feasible steady-state solution space of a network, its ability to predict dynamic time-courses for given experiments is limited [12]. Simulations from discrete-level models (e.g. Boolean) can be difficult to interpret due to sensitivity of model predictions to temporal updating schemes [13], assignment of discrete activity-levels to continuous-valued variables like concentration [14], and the limited ability to describe realistic timescales [15]. The tradeoffs inherent in many of these logic-based modeling approaches has recently been reviewed [16]. In addition, these modeling approaches are generally not compatible with the wealth of systems analysis tools for differential equations from control theory and dynamical systems. Piecewise-linear differential equation models overcome some of these limitations by making both species values and time continuous, but steady-state species activities are still binary [9,15,17]. Others have modeled signaling networks with continuous approximations of Boolean functions [18] that are implemented to minimize steady-state differences between Boolean and continuous models.

To address these limitations, we developed a normalized-Hill differential equation modeling approach that combines advantages of both biochemical and Boolean models. This approach uses normalized Hill functions and logical AND and OR operators to describe network crosstalk. We used this approach to model the cardiac β-adrenergic signaling pathway and performed a direct comparison with a previously validated biochemical model of the same network [6,7]. We then used this model to gain insight into the roles of feedback and feed-forward loops in the β-adrenergic pathway and examined potential crosstalk with integrin-mediated mechanotransduction. The analysis presented here demonstrates that the normalized-Hill differential equation modeling approach can provide reasonably accurate predictions of signaling properties, even when little parameter data is available.

## Results

### Toy signaling network

**Figure 1:**
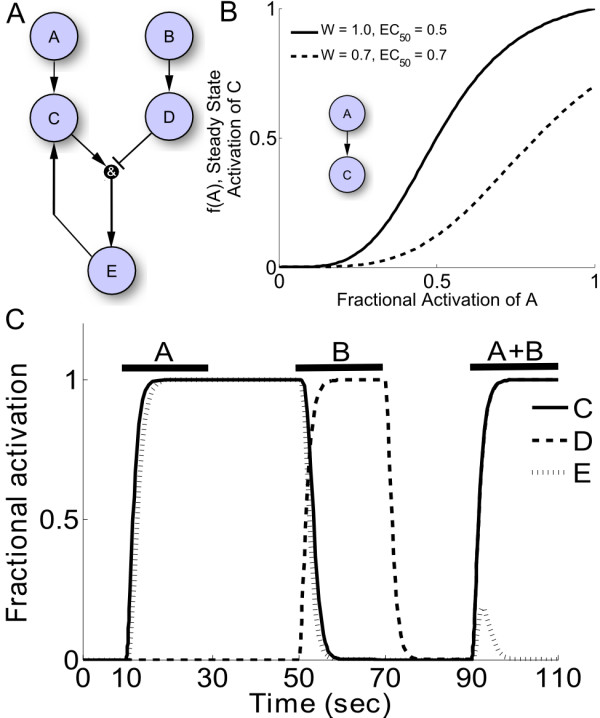
**Normalized-Hill toy network model**. A) Schematic of the 5-species toy network, including two inputs, an AND reaction, and a positive feedback loop. B) Characteristics of sample normalized-Hill functions (n = 4 for both curves). C) Simulated signaling dynamics in response to transient inputs A, B, and A + B. Output E remains active after removal of input A due to a bistable positive feedback loop, though this memory is erased with addition of B. Addition of both stimuli at the same time transiently activates output E. Parameters used are: τ = 1, W = 1, n = 1.4, and EC_50 _= 0.5.

For demonstration, we created a toy signaling network using our normalized-Hill differential equation approach. This simple network consists of two input ligands ("A" and "B") that activate receptors "C" and "D", respectively. A positive feedback loop exists between "C" and "E" that is inhibited when "D" is activated (see Figure 1A). The state variables represent the "fractional activation" of the signaling species, which is normalized to the maximal possible activity. Fractional activation varies continuously with time and can take on any value between 0 and 1, inclusive. For example, fractional activation for a substrate that is active only when phosphorylated is equivalent to the ratio of phosphorylated to total protein.

Model equations for this toy network are provided in "Methods" and Additional File 1, Supplemental Methods, though properties of our modeling approach are discussed here. Interactions between species are modeled using normalized Hill functions with 3 reaction parameters: the reaction weight "W", half-maximal effective concentration "EC_50_", and Hill coefficient "n". The reaction weight determines how much a given interaction activates or inhibits an output species and can take on any value between 0 and 1, inclusive. EC_50 _is the fractional activation of an input species required to induce half-maximal activation of an output species. Lastly, the Hill coefficient determines sensitivity to changes in inputs. The normalized Hill functions are constrained to f(0) = 0, f(1) = 1 and f(EC_50_) = 0.5. Additionally, species activities are controlled by the parameters τ and Y_MAX_, which are the reaction time constant and species maximal fractional activation, respectively. While Y_MAX _= 1 is typical, this value can be altered to reflect a change in protein expression relative to a reference condition. Typical default reaction and species parameter values are W = 1, EC_50 _= 0.5, n = 1.4, τ = 1, and Y_MAX _= 1; choice of default values for EC_50 _and n are examined in more detail below. Crosstalk between species is modeled using continuous functions analogous to Boolean AND and OR operations (see Methods).

While default parameters can provide qualitatively reasonable results, the parameters involved in these Hill functions can be directly measured in cellular experiments to quantitatively refine model predictions. For example, in the toy model, W_D_, K_B _and n_B _could all be determined directly from a single steady-state concentration response where B is varied and D is measured. Likewise, τ_D _could be determined experimentally by measuring the dynamic response of D in response to a step in B. If D is a kinase substrate, typical experiments could include quantitative Western blots, cellular immunofluorescence, or live-cell FRET biosensors similar to those used previously for measuring active PKA dynamics in cardiac myocytes [19].

Figure 1C shows a sample simulation of toy network signaling dynamics in response to a transient exposure to input "A", input "B", followed by both inputs simultaneously. Note that "C" and "E" retain full activity even after input "A" has been removed due to the presence of a bistable positive feedback loop. However, this memory is erased once input "B" is activated. When "A" and "B" are activated simultaneously, "E" is transiently activated but switches off once precursors "C" and "D" become highly active.

**Figure 2:**
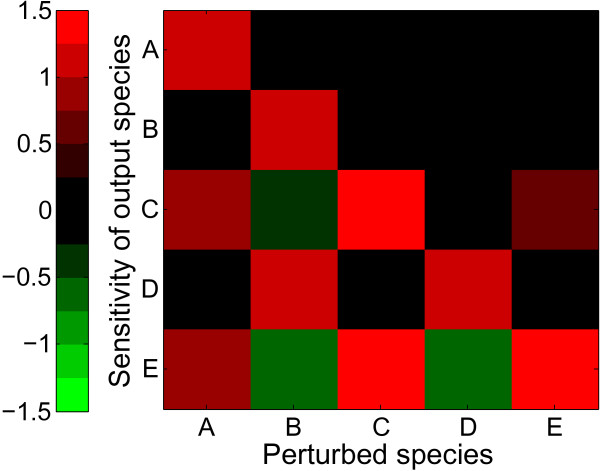
**Sensitivity analysis of the toy network**: Each column of the sensitivity matrix represents a numerical experiment in which one species was perturbed by -25%. The steady-state sensitivities of all model species were computed according to S = (ΔY/ΔP)(P_o_/Y_o_). The model predicts that E is positively regulated by A and C but negatively regulated by B and D.

Because this modeling approach uses ordinary differential equations, well-established systems approaches, such as quantitative sensitivity analysis, can be readily applied to study network relationships. The sensitivity plot shown in Figure 2 quantifies the global functional relationships between all species in the system. Each column simulates an individual experiment in which the maximum activity of one species is perturbed (e.g. "A_MAX_") and the subsequent changes in all network species' activities are quantified. The normalized steady-state sensitivities of these species to parameter perturbations are quantified according to S = (ΔY/ΔP)(P_o_/Y_o_) (see *Methods *for details). Graded positive and negative sensitivities are represented with shades of red and green, respectively. For example, the "B" column indicates that increased fractional activation of "B" causes increased activity of itself and "D", decreased activity of "E" and (to a lesser extent) "C", and no change in the activity of "A". This type of quantitative sensitivity analysis is not possible with many other logic-based or topological approaches.

### β-adrenergic signaling network

**Figure 3:**
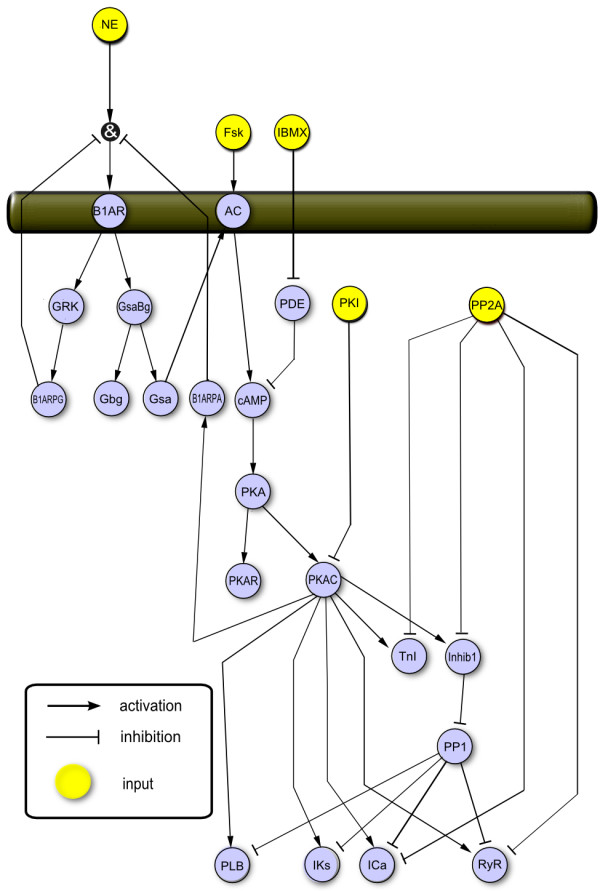
**Schematic of normalized-Hill β-adrenergic signaling network**. The normalized-Hill model consists of 25 species and 36 reactions. While developed using a normalized-Hill differential equation approach, the network topology closely mimics a well-validated biochemical model [6] to allow for direct comparisons.

We next applied our normalized-Hill modeling approach to the cardiac β-adrenergic signaling network, the major pathway that regulates contractility, metabolism, and gene regulation of the heart [20]. Following stimulation by β agonists (e.g. norepinephrine or NE), the β-adrenergic receptor couples with G proteins, which subsequently activate adenylyl cyclase (AC). AC synthesizes the second messenger cAMP, which causes dissociation of the regulatory and catalytic subunits of protein kinase A (PKAR and PKAC, respectively). PKAC phosphorylates several substrates, including phospholamban (PLB), the ryanodine receptor (RyR), troponin I (TnI), and inhibitor-1 (Inhib1), resulting in enhanced cardiac contractility. Figure 3 shows a schematic of this model, which includes 25 species and 36 reactions. All interactions were implemented using the same default parameters used in the toy signaling network. Importantly, the network topology for this normalized-Hill model was designed to be very similar to an extensively characterized and validated biochemical model of this same network [7].

**Figure 4:**
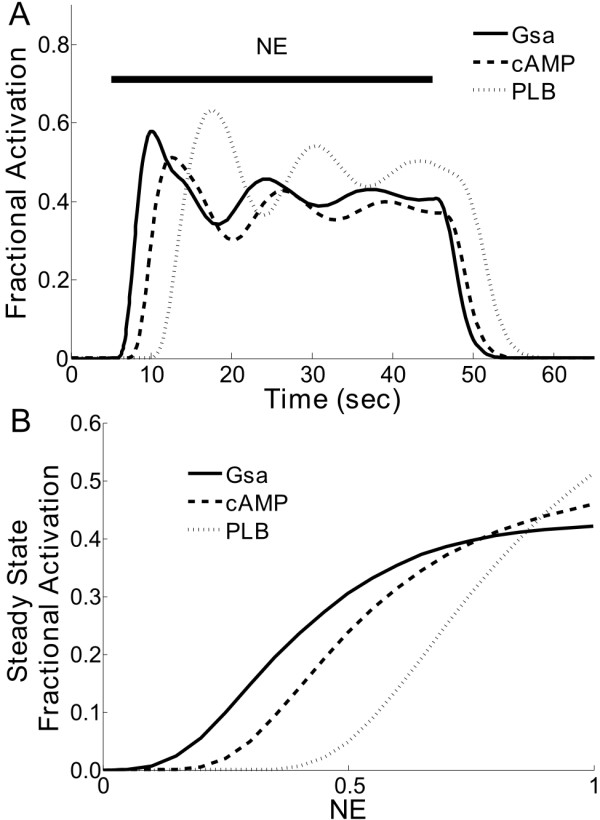
**Predicted dynamics and dose responses of the normalized-Hill β-adrenergic model**. A) Predicted dynamics of G proteins (Gsα), cyclic AMP (cAMP), and phospholamban (PLB) phosphorylation in response to a transient norepinephrine (NE) exposure (NE = 1). B) Steady-state concentration responses of Gsα, cAMP and PLB to varying levels of NE.

To test the normalized-hill β-adrenergic model, we simulated a simple experiment in which NE is added to the system and subsequently removed. The addition of NE indirectly activates G proteins (Gsα), cAMP, and PLB (Figure 4A). The response delay of cAMP and PLB to NE (as compared to Gsα) occurs because these species are farther downstream in the signaling cascade. An adaptive response to the sustained NE input is observed in these activation profiles, as seen experimentally for cAMP [21,22], which may be attributed to the negative feedback loops consisting of PKAC and GRK phosphorylation of β_1_ARs (see below). Responses for individual species activities during simulations with varying levels of NE were also evaluated; a sample dose response is shown in Figure 4B, where increased levels of NE produced concomitant increases in the fractional activities of Gsα, cAMP, and PLB, as expected from experiments [23,24].

### Role of feedback and forward loops in shaping network dynamics

**Figure 5:**
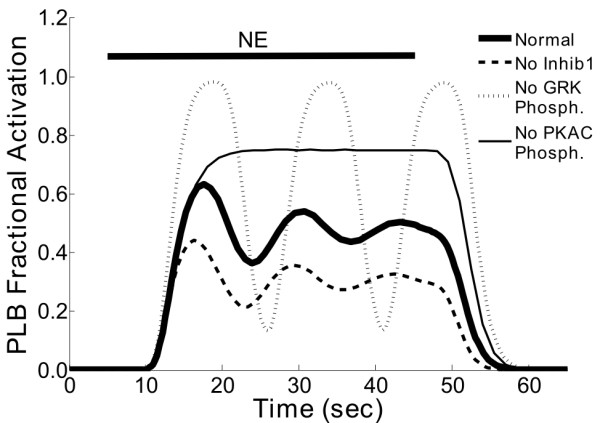
**Dynamic roles for feedback and feed-forward loops in the β-adrenergic network**. Time courses of PLB activation in response to transient NE exposures (NE = 1) with several network perturbations. The thick solid line is the response of the default model, showing moderate PLB activation and damped oscillations. Also shown are simulations in which either the inhibitor-1 coherent positive feed-forward loop (thick dashed line; W_PKAC-Inhib1 _= 0), the GRK negative feedback loop (thin dashed line; W_GRK-B1ARPG _= 0) or the PKAC negative feedback loop (thin solid line; W_PKAC-B1ARPA _= 0) were eliminated. Inhibitor-1 amplified PLB activation without affecting PLB dynamics. Both GRK and PKAC feedback loops decreased PLB phosphorylation via β_1_-adrenergic receptor desensitization, though PKAC controlled steady-state adaptation while GRK feedback dampened the PLB response.

Feedback and feed-forward loops are key network motifs that can enhance information processing by altering signaling dynamics or providing adaptation [25]. In this network, there are two negative feedback loops, both involving receptor phosphorylation by either PKA or G-protein receptor kinase (GRK), which desensitize the β-adrenergic receptor to ligand inputs. Receptor desensitization leads to an adaptive response to the ligand NE. For 4 PKA substrates (PLB, IKs, ICa, RyR) there are coherent positive feed-forward loops formed when PKA enhances substrate phosphorylation both directly (PKA phosphorylates the substrate) and indirectly by blocking inhibitor-1 (Inhib1), which phosphorylates protein phosphatase 1 (PP1), inhibiting substrate phosphorylation. We hypothesized that these feedback and feed-forward loops contributed to the predicted adaptive dynamics seen in our default simulations of PLB activity (see Figure 4A). To test this, we performed simulations in which each of these feedback or feed-forward loops were disrupted (i.e. setting their corresponding reactions weights, W_GRK-B1ARPG_, W_PKAC-B1ARPA_, or W_PKAC-Inhib1_, to zero) (Figure 5).

Surprisingly, disrupting individual loops had dramatically different consequences. Inhibition of the GRK negative feedback loop resulted in sustained oscillations, indicating that GRK negative feedback contributed to damping PLB phosphorylation. In contrast, inhibition of the PKA negative feedback loop raised the steady-state PLB activity and disrupted oscillations, showing that this feedback loop controlled steady-state PLB adaptation. Lastly, blocking the inhibitor-1 feed-forward loop reduced PLB activity without qualitatively affecting the timecourse, suggesting that this loop amplifies PLB signaling. Previous experimental and modeling studies comparing GRK and PKA feedback loops studied the β_2_-AR isoform, where receptor desensitization was driven primarily by GRK [26,27]. However, steady-state measurements in cells expressing β_1_-AR [28], the receptor isoform considered here, are consistent with the current model predictions that both GRK and PKA feedbacks contribute significantly to PLB responses, via β_1_-adrenergic receptor desensitization. Thus, the normalized-Hill modeling approach can be used to assess how various network architectures drive signaling dynamics, though results can be refined when experimental data is available (discussed further in subsequent sections).

### Quantitative sensitivity analysis of the β-adrenergic network

**Figure 6:**
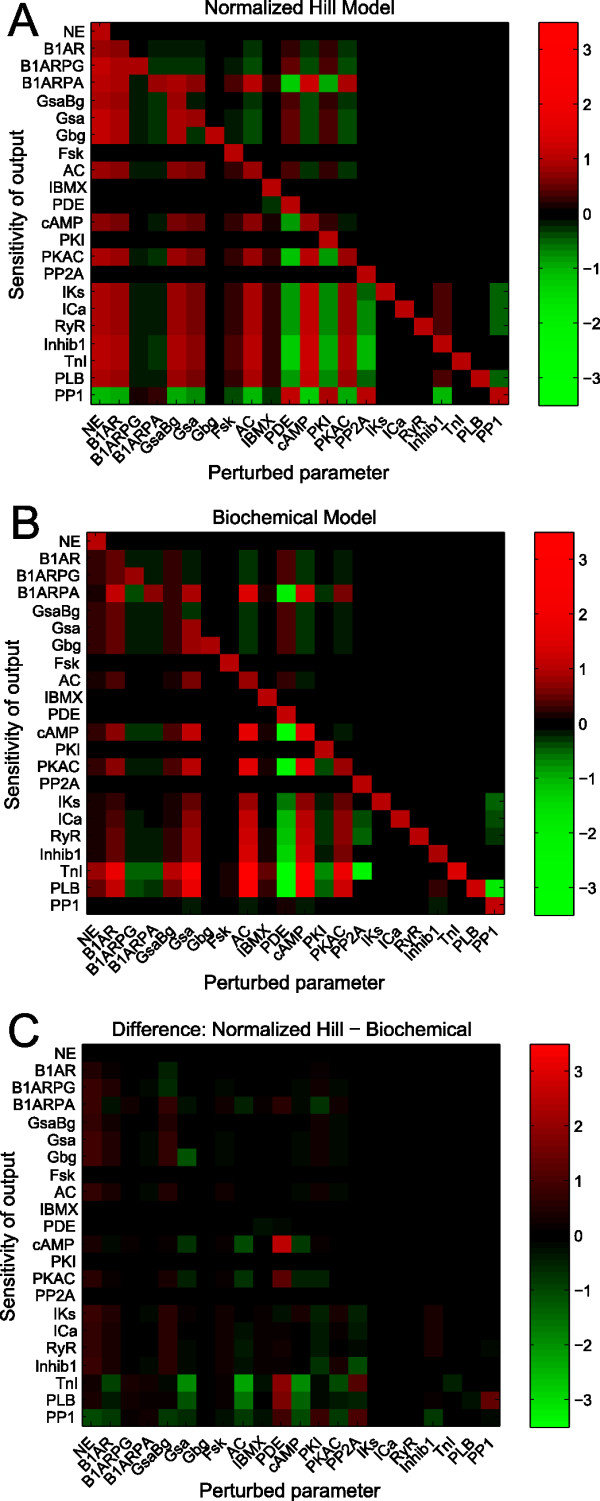
**Sensitivity analysis of the β-adrenergic network identifies key system regulators**. A) Sensitivity analysis of the normalized-Hill β-adrenergic model. Note the bright green squares prevalent in the phosphodiesterase (PDE), protein kinase inhibitor (PKI), and phosphatase 2A (PP2A) columns, implicating the importance of these three species as key negative regulators of the system. B) Sensitivity analysis of a detailed biochemical model of the same pathway, allowing for direct comparison of the two modeling approaches. A Pearson correlation coefficient of 0.75 was computed between the individual sensitivities of the two models, demonstrating substantial quantitative agreement between the functional outputs of the two modeled networks.

Quantitative sensitivity analysis provides an approach to systematically survey the functional relationships within a signaling network, often revealing unexpected systems properties. We performed sensitivity analysis on our normalized-Hill β-adrenergic model using the same approach as that implemented for our toy network (Figure 6A). For this analysis, model inputs were set at moderate activity levels to avoid saturation of the network's dynamic range (NE = 0.5, Fsk = 0.2, IBMX = 0.2). Species are generally ordered from upstream (top/left) to downstream (bottom/right) in the sensitivity matrix. From this analysis, several global properties of the network can be identified. The diagonal represents self-activation, which is not always prominent due to the negative feedback loops discussed earlier. While upstream species in the left portion of the matrix (NE through PKAC) affect many others, species further downstream in the pathway (PP2A through PP1) affect fewer. This indicates that the downstream species are in "branches" of the pathway and do not significantly feedback on upstream components.

Importantly, this analysis reveals quantitative relationships that are not apparent from the network topology alone. Several inhibiting (green) relationships, mainly involving PDE, PKI, and PP2A, are quantitatively prominent. These proteins appear to be key negative regulators of this network that may serve as potential therapeutic targets. PDE strongly inhibits (green) PKAC, TnI and B1ARPA, while it modestly activates B1AR and AC. These effects are not explained completely by path length, as PDE has a smaller effect on its direct target (cAMP) than species which are three steps away (e.g. TnI, B1ARPA). Indeed, the PDE inhibitor milrinone has been used for patients with congestive heart failure [29]. However, milrinone actually worsens mortality by elevating occurrences of ventricular arrhythmias [30,31], perhaps due to the large number of PDE-sensitive species suggested by our modeling results. PP2A is a strong inhibitor of TnI with less strong inhibition of IKs and PLB. In contrast, other perturbations such as Fsk and IBMX exhibit rather modest effects on the network despite affecting many other species in a qualitative sense.

Examining a particular row in this matrix allows one to identify perturbations that are more or less likely to affect a given output. For example, the calcium channel ICa appears very sensitive to activation by cAMP or inhibition by PDE, but is less sensitive to Fsk activation (same path length as PDE) or direct PP1 inhibition. Some species have low quantitative sensitivity even though they are within the same pathway. For example, direct substrates of PKAC (e.g. β_1_ARPA, ICa, PLB) are highly sensitive to perturbations in AC, cAMP and PKAC, while other species in the same pathway, such as β_1_AR or Gsα, are much less sensitive. Many of these quantitative predictions cannot be achieved by qualitative graph analysis, and may be used to prioritize future experiments.

### Direct comparison of normalized-Hill and biochemical models

In order to assess the predictive accuracy of the normalized-Hill β-adrenergic model, the model's sensitivity matrix was compared to a similar matrix generated from a detailed biochemical model of the same signaling network [7] (Figure 6B, additional details in *Methods*). The difference between these two matrices is shown in Figure 6C. Overall, there are a number of similarities between the structures of the two sensitivity matrices. Note the clear divisions between upstream and downstream components, indicating similar predictions of global functional relationships across the two models. To quantify these similarities, we computed the Pearson correlation coefficient between the corresponding sensitivities of the two models and obtained a value of 0.75. This analysis indicates that there is substantial quantitative agreement between the two models, considering the significant differences in their formulation and the use of default parameters in the normalized-Hill model.

To test the appropriateness of our default selections for Hill coefficients and EC_50 _values, we examined correlation coefficients between the biochemical model and normalized-Hill models while varying default "n" and EC_50_. We found that the predictions were insensitive to the choice of default Hill coefficient (though n = 1.4 was optimal), but highly sensitive to the intuitive EC_50 _value of 0.5. Higher sensitivity to EC-_50 _arises because a linear pathway tends to systematically amplify or diminish signals when default EC-_50 _deviates from 0.5 (see Additional File 2, Figure S1).

Despite striking similarities between predictions by the normalized-Hill and biochemical models, there are also several notable discrepancies that may provide further insight. To highlight these, we re-classified the predicted sensitivities from both models as either "activating" (S = 1), "inhibiting" (S = -1), or "neutral" (S = 0) and produced qualitative sensitivity matrices using only these values (see Additional File 3, Figure S2, additional details in Methods). Globally, the two models showed good agreement in terms of individual sensitivity types, with 457 out of 484 (94%) individual sensitivities qualitatively matching. Of the 27 mismatches, 3 of these were in opposite directions (0.62% of the total). This was seen, for example, when Gsα/βγ (GsaBg) was perturbed: the normalized-Hill model predicted that reduction of Gsα/βγ increases activation of B1AR and B1ARPG, while the opposite result was obtained from biochemical model predictions. In the normalized-Hill model, reduced PKA feedback via reduced GsaBg enhanced fractional activation of B1AR and, thus, B1ARPG. These results are expected given that all normalized-Hill model interactions are unidirectional. On the other hand, the biochemical model uses detailed mass action kinetics to describe these interactions, where a reduction in total Gsα/βγ can actually pull additional free receptors to a bound form, resulting in reduced B1AR and B1ARPG. Thus, there are competing mechanisms in this portion of the network (PKA feedback versus G-protein activation) that are represented differently between the two model types. Related to this issue, most species in the biochemical network are sensitive to perturbations in G_βγ_, though to a very small extent quantitatively (compare Gbg columns in Figures 6B and S2). This subtle difference arises because G_βγ _is a terminal node in the normalized-Hill model, whereas the additional details of the biochemical model allow G_βγ _perturbations to very modestly influence downstream signaling. Though quantitatively insignificant in most cases, these results highlight subtle limitations in the normalized-Hill β-adrenergic signaling model that can be addressed with additional reactions (though not done here).

Other discrepancies are attributed to differences in the extent to which spatial compartmentation was incorporated in the two models. For example, all downstream PKA substrates are sensitive to inhibitor-1 perturbation in the normalized-Hill model since this species inhibits global PP1 activity. In the biochemical model, however, inhibitor-1 is only responsible for local inhibition of PP1 near PLB (but not other PKA substrates; see Inhib1 columns in Figure 6). Thus, many of the discrepancies can be attributed to subtle differences in network connectivity rather than the modeling approaches themselves.

Other logic-based modeling approaches have also used logical AND/OR interactions and differential equations to describe biochemical networks [9,15,18]. While these logic-based approaches have not previously been directly compared to a biochemical model, we extended the sensitivity analysis (as in Figure 6) to examine these approaches as well. The β-adrenergic model was re-implemented using piece-wise linear, Hill, or linear activation functions. As shown in Additional File 4, Figure S3 and Additional File 5, Figure S4, the normalized-Hill approach exhibits substantially better performance compared with the piece-wise linear approach or Hill equations with previously-used default parameters (n = 3, K = 0.3) [18]. The poor performance of the piece-wise linear approach is largely due to the fact that its steady-state values are restricted, hindering predictions of sensitivity to a quantitative perturbation. The Hill approach performed poorly as well, but its performance could be improved somewhat by optimizing parameters "n" and "K". Linear activation functions worked fairly well for this steady-state sensitivity analysis, but linear activation functions are not able to predict nonlinear phenomena such as the bistability shown in Figure 1.

**Figure 7:**
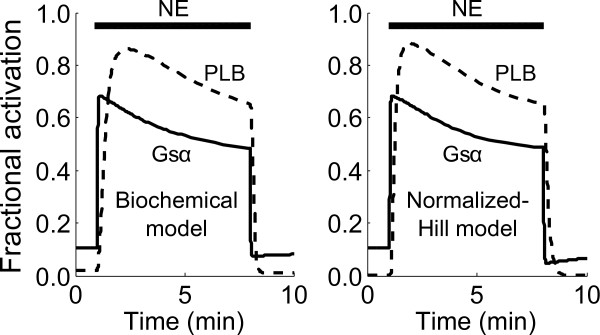
**Comparing predicted dynamics of the normalized-Hill model with a detailed biochemical model**. The left panel shows normalized dynamic responses of Gsα and PLB phosphorylation predicted by the biochemical model during a transient 10 nM NE exposure. Normalized-Hill model predictions of the same outputs during a similar NE exposure are shown in the right panel. Activity levels and dynamics of the normalized-Hill model traces were fit to those of the biochemical model via least-squares parameter estimation. In all, 11 parameters were adjusted (4 τ's, 3 W's, 3 EC_50_'s, and an additional basal receptor activity term) to mimic biochemical model outputs using the normalized-Hill model.

To further compare predictions from the normalized-Hill and biochemical models, we examined the predicted dynamics of Gsα and PLB in response to a transient NE exposure. Though global functional relationships are strikingly similar between the two models (Figure 6), the normalized-Hill model outputs using all default parameters contains damped oscillations that are not prominent in the biochemical model (compare Figure 4A with the left panel in Figure 7). To test whether the normalized-Hill model predictions can be refined further, we fit several parameters in the normalized-Hill model to time-course data from the biochemical model using a nonlinear least squares optimization algorithm (lsqnonlin in Matlab). Eleven parameters (4 τ's, 3 weights, 3 EC_50_'s, and an additional basal receptor activity term) were adjusted to fit model predictions (see Additional file 1, Supplemental Methods).

These parameter adjustments allowed for more similar signaling dynamics and comparable peak fractional activities of Gsα and PLB compared with the biochemical model (Figure 7), with more gradual adaptation rather than damped oscillations. We further probed whether conclusions drawn regarding the feedforward and feedback loops from Figure 5 would be maintained in the adjusted model. As shown in Additional File 6, Figure S5, while the PLB response exhibits gradual adaptation rather than damped oscillations, the role of the feedforward and feedback loops are similar: Inhibitor 1 amplifies PLB, B1ARPG attenuates PLB, and B1ARPA dominates the degree of steady-state adaptation. These analyses demonstrate that the normalized-Hill model largely captures key features of the more detailed biochemical model, that specific model discrepancies can be quantitatively explained by parameter differences, and that dynamic predictions can be refined by fitting relevant parameters to available data.

### Model extension to incorporate integrin-mediated mechanotransduction

**Figure 8:**
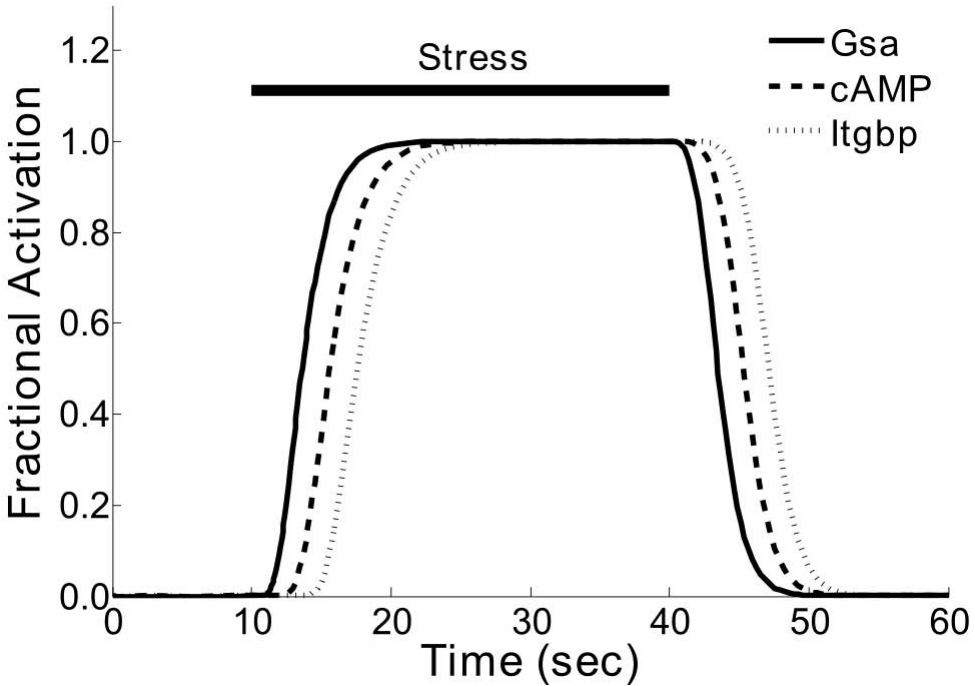
**Model extension to include integrin activation and phosphorylation in response to mechanical stress**. Reactions for stress-induced integrin activation and subsequent phosphorylation by PKA were identified from recent protein interaction data http://www.pathwaycommons.org/pc/. Shown are predicted dynamics during a transient mechanical stress stimulus for Gsα (which is stimulated by the stress input), cAMP, and integrin phosphorylation (Itgbp) by PKA. The mechanisms for this additional pathway are schematized in Additional File 7, Figure S6.

Due to the wealth of data needed to parameterize a biochemical model, it can be difficult to extend the model with new experimental findings. However, the normalized-Hill modeling framework may be more easily extended. We searched the Pathway Commons database http://www.pathwaycommons.org/pc/ for additional PKA substrates, finding 46 pathways and 63 catalysis reactions. The first listed interaction was PKA-mediated phosphorylation of β-integrins, which regulates cellular adhesion [32]. Alenghat *et al. *recently showed that shear stresses applied to β_1 _integrins using RGD-coated magnetic microbeads stimulated cAMP via Gsα [33]. By integrating this new data into our model, we predicted that mechanical stresses may activate the cAMP/PKA pathway in cardiac myocytes in a manner independent of β-adrenergic receptors. Additional File 7, Figure S6 shows how these mechanisms were incorporated into the normalized-Hill model. We ran a simulation in which only mechanical stress was applied to the network (i.e. NE = Fsk = IBMX = 0), and saw activation of Gsα, cAMP, and integrin β-subunit phosphorylation (Itgbp) (Figure 8). Note that the response times of these outputs to the stress stimulus are dependent on their relative positions in the signaling cascade. As an independent validation of these predictions, prior work has shown that stretch of intact heart induces a rise in cAMP, which may contribute to stretch-induced increases in cardiac contractility [34].

## Discussion and conclusions

We developed a normalized-Hill differential equation modeling approach that combines advantages of both biochemical and Boolean models. Even when parameters are not available, this approach allows for predictions of signaling dynamics and is compatible with a wide range of existing systems analyses, including quantitative sensitivity analysis. Furthermore, these models can be iteratively refined by either tuning parameter values (as done here) or adding additional reactions to better reflect quantitative features of experimental data. This approach was evaluated by direct quantitative comparison with a well characterized and experimentally validated biochemical model of the cardiac β-adrenergic signaling network [7].

The analysis revealed several new insights into relationships between β-adrenergic network topology and dynamics. Dissection of the feedforward and feedback loops showed that each loop could play a unique role in regulating PLB phosphorylation dynamics. The Inhibitor-1-mediated coherent positive feedforward loop was predicted to amplify signaling, which is consistent with reports of decreased PLB phosphorylation in mice with Inhibitor-1 ablation [35]. In contrast, PKA- and GRK-mediated negative feedback loops (B1ARPA and B1ARPG, respectively) reduced PLB phosphorylation either by enhancing steady-state adaptation (PKA) or attenuating the overall PLB signal (GRK). While the kinetics and damped oscillations were sensitive to the rate constants for these feedback loops, the overall roles of the feedback loops were maintained using both default and adjusted parameters in the normalized-Hill model. Quantitative sensitivity analysis revealed the global structure of functional relationships in this network, which was not clear from previous analyses of the biochemical model [6]. This suggests that therapeutically targeting species within the PKA negative feedback loop may be less specific than targeting individual branches involving PKA substrates. Sensitivity analysis highlighted key hubs (such as PDE's) as major inhibitors of this pathway, consistent with drug development in this area [36]. Sensitivity analysis also revealed quantitative relationships that are not seen from the topology alone, such as lower sensitivity of Gsα and AC to perturbations in PKAC. Finally, model extension allowed analysis of crosstalk with integrin-mediated mechanotransduction, helping to explain experimentally-observed cAMP synthesis and increased cardiac contractility during stretch [34].

The normalized-Hill differential equation approach is related to logic-based approaches that also attempt to simulate biological networks with limited parameter data. The most well-characterized qualitative approach is Boolean network modeling, where species states are binary and the network is simulated over discrete time [37]. The binary state limitation can be partially overcome by allowing multiple discrete states [38], but this requires additional information to determine the number of states and to what state a given reaction activates a species. Discrete-time models can also require complex updating schemes to avoid artifacts [15], resulting in stochastic simulation data that increases the complexity of subsequent analysis. To address these issues, piecewise linear differential equations have been developed, which allow continuous-valued species states and continuous time [9,15,17]. Like our approach, piecewise linear differential equations use logical AND/OR operations and represent time-dependence with differential equations. However, this approach still uses discrete thresholds for activation of reactions that may not be consistent with experimental data, and the steady-state predictions of these models are binary [39]. As a result of limited possible steady-state values, we found that piecewise linear differential equations could not accurately predict quantitative model sensitivities. However, an advantage of the piecewise linear approach is that it requires fewer parameters: time constants and activation threshold parameters.

As opposed to discrete thresholds, a wide variety of biological networks exhibit smoothly saturating activation profiles that are well-approximated by Hill functions. Hill functions have been used to model a wide range of phenomena, including hemoglobin binding [40], dynamics of synthetic gene networks [41] and signaling pathways [42]. Because our state variables are generally limited to values between 0 and 1, we developed a normalization scheme that constrained activating Hill functions such that f(0) = 0, f(1) = 1 and f(EC_50_) = 0.5. We found that normalizing with just 2 of these 3 constraints, as done previously [18], caused undesirable shifts in EC_50_'s or maximal activities, resulting in either diminished or enhanced signaling down a linear pathway. These artifacts limited the dynamic range of the pathway and hindered quantitative sensitivity analysis as compared with the biochemical model. In addition, we have included parameters that allow for further quantitative refinement of model predictions: reaction weights (W) and maximum activity values (Y_MAX_). Reaction weights allow certain reactions to exert more influence than others. This was important for refinement of our β-adrenergic model because only partial receptor desensitization is seen experimentally [22]. Ability to perturb Y_MAX _values was critical for performing quantitative sensitivity analysis in the present work, but this also allows for future incorporation of changes in protein expression that are independent of the level of fractional activation.

Direct quantitative comparison of the normalized-Hill β-adrenergic model with a validated biochemical model allowed characterization of the strengths and weaknesses of this approach in a controlled environment. To our knowledge, such a direct comparison has not been previously made for other logic-based approaches. Through sensitivity analysis and time course comparisons, we found that default parameter values were sufficient to predict global functional relationships with relatively high quantitative agreement, especially in terms of responses to systematic perturbations (correlation coefficient of 0.75). Indeed, the vast majority of experimental data on signaling networks provides relative rather than absolute quantification [43]. The normalized-Hill activation function outperformed piecewise linear, Hill and linear activation functions. Absolute quantitative predictions of particular time constants or signal magnitudes can be achieved with either prior parameter knowledge or optimization-based parameter estimation. Because only a small number of steps in a pathway are highly sensitive or rate-limiting, high quantitative agreement was obtained when fitting 11 parameters. By comparison, the biochemical model contains 88 parameters.

Because the model is composed of differential equations, this modeling framework is compatible with the wealth of analysis tools currently available from dynamical systems including sensitivity analysis (shown here), bifurcation analysis [44] and parameter estimation (performed in specific cases here) [45]. These models may also be directly integrated with multi-scale models of electrical-mechanical coupling or other aspects of physiology [46].

A number of differences from biochemical models must be considered regarding model structure. First, species states are quantified in terms of fractional activation rather than absolute quantities such as concentration. While this may be appropriate for proteins where the data is often normalized and the focus is on post-translational modifications, small molecules such as cyclic AMP are always in an "active" form. For such cases, fractional activation may be considered to be relative to the amount measured under a highly-stimulated condition. Second, not all relationships are well-represented by activating or inhibiting Hill functions. Other activation functions can be used as appropriate, and the use of differential equations allows a detailed biochemical module to be easily embedded within a larger normalized-Hill model or vice versa. Third, this normalized-Hill approach does not explicitly incorporate competitive inhibition, leading to a small number of incorrect predictions when G_βγ _was perturbed. Finally, multiple post-translational modifications on a single protein may be required to be represented as separate species, as we have done for the GRK and PKA phosphorylation sites on the β-adrenergic receptor (B1ARPG and B1ARPA, respectively).

Despite these limitations, the normalized-Hill differential equation modeling approach predicts quantitative systems properties that typically require detailed biochemical models. Using default parameters, we obtained accurate simulations of the β-adrenergic signaling network that revealed unexpected functional relationships, generated experimentally testable predictions and readily incorporated new parameter and reaction data. This suggests that this approach may facilitate larger scale reconstruction and analysis of signaling networks, particularly for those where biochemical characterization is limited.

## Methods

### Normalized-Hill differential equation modeling approach

Species dynamics are predicted using ordinary differential equations, where the state variables represent fractional activation of each species. Species interactions were defined with normalized activating or inhibiting Hill functions (f_act _or f_inhib_) which are described below. Pathway crosstalk was implemented using logical AND and OR operations: "f(x)f(y)" and "f(x)+f(y)-f(x)f(y)", respectively. Using this approach, the toy model described in Figure 1 was implemented with the following differential equations:

(1.1) $$\begin{array}{l} \frac{dD}{dt}=\frac{1}{\tau_{D}}\left(W_{BD}f_{act}(B)D_{MAX}-D\right)\\ \frac{dE}{dt}=\frac{1}{\tau_{E}}\left(W_{CDE}f_{act}(C)f_{inhib}(D)E_{MAX}-E\right)\\ \frac{dC}{dt}=\frac{1}{\tau_{C}}\left[\left(W_{AC}f_{act}(A)+W_{EC}f_{act}(E)-W_{AC}f_{act}(A)W_{EC}f_{act}(E)\right)C_{MAX}-C\right] \end{array}$$

where *τ *is the time constant for a given species, W is the reaction weight (constrained to 0≤W≤1), and Y_MAX _is the maximal fractional activation allowing simulations of knock-down (Y_MAX _< 1) or overexpression (Y_MAX _> 1). The normalized activating or inhibiting Hill functions, depicted graphically in Figure 1B, have the following form:

(1.2) $$f_{act}(X)=\frac{BX^{n}}{K^{n}+X^{n}};f_{inhib}(X)=1-\frac{BX^{n}}{K^{n}+X^{n}}$$

where B and K are constrained such that f_act_(0) = 0, f_act_(EC_50_) = 0.5 and f_act_(1) = 1. From these constraints, we derived that:

(1.3) $$B=\frac{EC_{50}{}^{n}-1}{2EC_{50}{}^{n}-1};K={\left(B-1\right)}^{1/n}$$

We further constrained f_act_(X) = 1 for X≥1 to ensure that species activities are limited to Y_MAX_. As default parameters, we used W = 1, EC_50 _= 0.5, n = 1.4, τ = 1, and Y_MAX _= 1. Sensitivity of β-adrenergic model predictions to the choice of default values for n and EC_50 _were examined (see Additional File 2, Figure S1). The model was insensitive to the default Hill coefficient, with Pearson correlation coefficients ≥ 0.93 for 1.01 ≤ n ≤1.7 compared to n = 1.4. Sensitivity to default EC_50 _was higher because a value other than 0.5 causes a linear pathway to systematically amplify or diminish signaling at subsequent steps. All differential equations were implemented in MATLAB (MathWorks, Natwick MA) and solved numerically with the ode23 function.

### Sensitivity analysis

Two-dimensional sensitivity matrices were generated from normalized-Hill model simulations according to the equation S_ij _= (ΔY_i_/ΔP_j_)(P_o,j_/Y_o,i_), where *S_ij _*is the sensitivity of species "i" to perturbation of species "j", ΔY_i _is the change in steady-state output of species "i", and ΔP_j _is the change in parameter "j". These sensitivities are normalized to the original output (Y_o,i_) and parameter value (P_o,j_) to facilitate comparison between parameters and species. For a given numerical experiment (i.e. a column in a sensitivity matrix), a single species' Y_MAX _was perturbed by *ΔP *(in general, -25%). The model was run to steady state and the normalized sensitivities in all model species (rows of the sensitivity matrix) were computed. Thus, the sensitivity matrix represents a total of n_i_*n_j _predictions from n_j _numerical experiments. We found that different perturbation magnitudes (-10 to -75%) produced very similar sensitivity matrices (correlation coefficient > 0.98).

### Comparison of normalized-Hill model with biochemical model

Normalized-Hill model predictions were compared to our previously described biochemical model [7] in terms of parameter sensitivities and activation dynamics. Sensitivity analysis was computed as described for the normalized-Hill model, except that the biochemical model does not incorporate Y_MAX _parameters. Therefore, the most analogous parameters were perturbed: total species concentrations (where explicitly defined) or catalytic rate constants associated with production of a particular output. Biochemical model outputs were also selected to be consistent with the normalized-Hill model species as closely as possible. For quantitative comparison of normalized-Hill and biochemical model sensitivity matrices, a Pearson correlation coefficient was calculated from the individual elements of the sensitivity matrices. For a more qualitative comparison of the two models, we classified individual elements of the sensitivity matrix as either "activating" (S = 1), "inhibiting" (S = -1) or "neutral" (S = 0), in which threshold sensitivities of ±0.003 (determined by visual inspection of the histograms of individual sensitivities from the two models) were used to reassign individual values. Comparison of normalized-Hill and biochemical models was performed by counting the number of qualitatively matching individual sensitivities and dividing by the total number of matrix elements.

Biochemical model activation time courses were performed in similar fashion to normalized-Hill simulations. Reported values are normalized to the maximal activation level for that particular output (e.g. concentration of phosphorylated PLB subunits over total PLB). Ligand concentration was set to 10 nM where 'NE' is labeled in figures depicting biochemical model outputs.

In some simulations (Figure 7), normalized-Hill model parameters were adjusted to match outputs from the biochemical model. A list of all adjusted parameters is provided in Additional file 1, Supplemental Methods, although the fitting procedure is described here. Time courses for Gsα and PLB phosphorylation were obtained from the biochemical model during a transient NE exposure and normalized to their maximum values (i.e. phosphorylated PLB over total PLB protein). Parameters in the normalized-Hill model were then optimized using nonlinear least-squares minimization (lsqnonlin in MATLAB). This function adjusts selected parameter values to minimize the sum of square errors between data points; in this case, errors between normalized-Hill and the normalized biochemical model predictions were minimized. Several rounds of fitting with randomized initial parameter guesses, along with some manual adjustments, produced the final set of parameters used to generate the normalized-Hill model curves in Figure 7. See Additional File 1, Supplemental Methods for further details.

## Authors' contributions

JS planned the project; MK and AS performed the analysis; MK, AS and JS wrote the manuscript. All authors read and approved the final version of the manuscript.

## Acknowledgements

The authors thank Dr. Jason Papin for critical review of the manuscript. This work was supported by National Institutes of Health grant HL094476 and the American Heart Association grant 0830470N.

## Supplementary Material

Additional file 1 **Supplemental Methods**. Model equations and description of parameter estimation methods. Click here for file

Additional file 2 **Figure S1: Dependence of model accuracy on choices for default parameters**. A) Varying the choice of default Hill coefficient has little effect on the predictive capability of the normalized-Hill model compared to the detailed biochemical model, though n = 1.4 is optimal. B) Strength of normalized-Hill predictions are very sensitive to the choice of default EC50, where model predictions dramatically worsened as default EC50 deviated from the intuitive value of 0.5. Click here for file

Additional file 3 **Figure S2: Qualitative comparison of sensitivity matrices from normalized-Hill and biochemical β-adrenergic models**. A) Individual elements of each sensitivity matrix were re-classified as "activating" (red), "inhibiting" (green) or "neutral" (black), in which a threshold sensitivity of +/- 0.003 was determined by visual inspection of a histogram of sensitivities for each model. B) Difference plot showing regions of qualitative discrepancy between normalized-Hill model and the biochemical model. Globally, the two models showed good agreement in terms of individual sensitivity types, with 457 out of 484 (94%) individual sensitivities qualitatively matching. Of the 27 mismatches, only 3 sensitivities were in opposite directions (difference of -2; 0.62% of the total, see main text for further details). Click here for file

Additional file 4 **Figure S3: Comparison of biochemical model with normalized-Hill model and alternative modeling implementations**. Differential equation models of the same β-adrenergic network were generated using linear, piece-wise linear, or traditional Hill activation functions. All other parts of the model were kept constant, including time constants, reaction weights, AND/OR logic, and network topology. A) Pearson correlation coefficients were computed by comparing the sensitivity matrix of each model against the sensitivity matrix of the biochemical model. The normalized-Hill approach produces the highest level of agreement. B, left) Sensitivity matrix using Hill activation functions of n = 3 and EC50 = 0.3, as used previously for default parameters a for T-cell signaling network [17]. B, right) Difference between the sensitivity matrices from the traditional Hill model (n = 3, EC50 = 0.3) and the biochemical model, indicating areas of discrepancy. Sensitivity matrices for remaining modeling approaches are shown in Additional File 5, Figure S4. Click here for file

Additional file 5 **Figure S4: Sensitivity matrices and differences from the biochemical model for β-adrenergic models implemented with "piece-wise linear", Hill, and linear activation functions**. A) Traditional Hill activation with n = 2 and EC_50 _= 0.5. B) Activation was implemented as f_act_(x < 0.5) = 0, f_act_(x≥0.5) = 1 as described for "piece-wise linear differential equations" in [9,15]. White indicates values with no Real solution (NaN), which were excluded from computation of the correlation coefficient. The poor agreement is related to the limited number of possible steady-state values using this activation function. C) Linear activation functions, implemented as f_act_(x) = x. Click here for file

Additional file 6 **Figure S5: Dynamic roles for feedback and feed-forward loops in the β-adrenergic network in the adjusted normalized-Hill Model**. Using the adjusted model described in Figure 7, perturbations to feedback and feed-forward loops were performed as in Figure 5. The thick solid line is the response of the adjusted model to transient NE exposure, showing moderate PLB activation and partial adaptation to constant input. Also shown are simulations in which either the inhibitor-1 coherent positive feed-forward loop (thick dashed line; WPKAC-Inhib1 = 0), the GRK negative feedback loop (thin dashed line; WGRK-B1ARPG = 0) or the PKAC negative feedback loop (thin solid line; WPKAC-B1ARPA = 0) were eliminated. Inhibitor-1 amplified PLB activation without affecting PLB dynamics. Both GRK and PKAC feedback loops decreased PLB phosphorylation via β1-adrenergic receptor desensitization, though PKAC controlled steady-state adaptation while GRK feedback attenuated the PLB response. While the adjusted model does not exhibit the damped PLB oscillations of the default model (Figure 5), the roles of these feed-forward and feedback loops were largely maintained. Click here for file

Additional file 7 **Figure S6: Schematic of the β-adrenergic network with addition of integrin signaling**. Based on recent protein interaction data http://www.pathwaycommons.org/pc/, reactions involving mechanical stress, RGD-coupled microbeads, integrins (Itg), and PKA-mediated integrin phosphorylation (Itgbp) were added to the model to demonstrate the extensibility of the normalized-Hill modeling approach. Click here for file
