# Supplementary material for: Modeling cardiac β-adrenergic signaling with normalized-Hill differential equations: comparison with a biochemical model
Source: BMC Syst Biol. 2010 Nov 18;4:157. doi: 10.1186/1752-0509-4-157 (PMC2993667; doi:10.1186/1752-0509-4-157)
Supplement: Additional file 1 — Supplemental Methods. Model equations and description of parameter estimation methods. [file 1752-0509-4-157-S1.PDF]

Supplemental Text for Kraeutler et al, "Modeling cardiac β-adrenergic signaling with normalized-Hill differential equations: comparison with a biochemical model"

Toy Normalized-Hill Model Equations:

$$\frac{dA}{dt} = \frac{1}{\tau_A}[W_A Y_{MAX}(A) - A]$$
$$\frac{dB}{dt} = \frac{1}{\tau_B}[W_B Y_{MAX}(B) - B]$$
$$\frac{dC}{dt} = \frac{1}{\tau_C}[OR(f_{act}(A), f_{act}(E))Y_{MAX}(C) - C]$$
$$\frac{dD}{dt} = \frac{1}{\tau_D}[f_{act}(B)Y_{MAX}(D) - D]$$
$$\frac{dE}{dt} = \frac{1}{\tau_E}[f_{act}(C)f_{inhib}(D)Y_{MAX}(E) - E]$$

where $f_{act}(X)$ is a normalized-Hill activation function of activating species "X" with reaction parameters reaction weight "W", Hill coefficient "n", and EC50. The equation takes on the form:

$$f_{act}(X) = W\frac{\beta X^n}{K^n + X^n}$$

where 'β' and 'K' are computed for each reaction according to:

$$\beta = \frac{EC50^n - 1}{2EC50^n - 1} \text{ and } K = (\beta - 1)^{1/n}.$$

Equations for β and K were derived using the constraints that $f_{act}(0)=0$, $f_{act}(EC50)=0.5W$, and $f_{act}(1)=W$.

f_{inhib} is a normalized-Hill inhibition function of inhibiting species "X" that takes in parameters W, n, and EC50 for each reaction. The equation takes on the form:

$f_{inhib}(X) = W - f_{act}(X)$

AND reactions are implemented as multiplicative combinations of activating and inhibiting functions (see differential for state variable E). OR functionality is computed according to:

OR(a,b) = a + b – a*b

Where 'a' and 'b' are activating or inhibiting functions of any species A and B (see differential for state variable C for sample implementation).

Normalized-Hill β-adrenergic model equations:

The differential equations for the normalized-Hill model of the β-adrenergic signaling network are listed below. Unless stated otherwise, default parameters were used: W = 1, EC_{50} = 0.5, n = 1.4, τ = 1, and Y_{MAX} = 1. The entire model consists of 25 state variables (species) controlled by 36 reactions.

$$\frac{dNE}{dt} = \frac{1}{\tau_{NE}}[W_{NE}Y_{MAX}(NE) - NE]$$

$$\frac{dB1AR}{dt} = \frac{1}{\tau_{B1AR}}[f_{act}(NE)f_{inhib}(B1ARPG)f_{inhib}(B1ARPA)Y_{MAX}(B1AR) - B1AR]$$

$$\frac{dGRK}{dt} = \frac{1}{\tau_{GRK}}[f_{act}(B1AR)Y_{MAX}(GRK) - GRK]$$

$$\frac{dB1ARPG}{dt} = \frac{1}{\tau_{B1ARPG}}[f_{act}(GRK)Y_{MAX}(B1ARPG) - B1ARPG]$$

$$\frac{dB1ARPA}{dt} = \frac{1}{\tau_{B1ARPA}}[f_{act}(PKAC)Y_{MAX}(B1ARPA) - B1ARPA]$$

$$\frac{dGsabg}{dt} = \frac{1}{\tau_{Gsabg}}[f_{act}(B1AR)Y_{MAX}(Gsabg) - Gsabg]$$

$$\frac{dGsa}{dt} = \frac{1}{\tau_{Gsa}}[f_{act}(Gsabg)Y_{MAX}(Gsa) - Gsa]$$

$$\frac{dGsbg}{dt} = \frac{1}{\tau_{Gsbg}}[f_{act}(Gsabg)Y_{MAX}(Gsbg) - Gsbg]$$

$$\frac{dFsk}{dt} = \frac{1}{\tau_{Fsk}}[W_{Fsk}Y_{MAX}(Fsk) - Fsk]$$

$$\frac{dAC}{dt} = \frac{1}{\tau_{AC}}[OR(f_{act}(GSA), f_{act}(Fsk))Y_{MAX}(AC) - AC]$$

$$\frac{dIBMX}{dt} = \frac{1}{\tau_{IBMX}}[W_{IBMX}Y_{MAX}(IBMX) - IBMX]$$

$$\frac{dPDE}{dt} = \frac{1}{\tau_{PDE}}[f_{inhib}(IBMX)Y_{MAX}(PDE) - PDE]$$

$$\frac{dcAMP}{dt} = \frac{1}{\tau_{cAMP}}[OR(f_{act}(AC), f_{inhib}(PDE))Y_{MAX}(cAMP) - cAMP]$$

$$\frac{dPKA}{dt} = \frac{1}{\tau_{PKA}}[f_{act}(cAMP)Y_{MAX}(PKA) - PKA]$$

$$\frac{dPKAR}{dt} = \frac{1}{\tau_{PKAR}}[f_{act}(PKA)Y_{MAX}(PKAR) - PKAR]$$

$$\frac{dPKI}{dt} = \frac{1}{\tau_{PKI}}[W_{PKI} Y_{MAX}(PKI) - PKI]$$

$$\frac{dPKAC}{dt} = \frac{1}{\tau_{PKAC}}[OR(f_{act}(PKA), f_{inhib}(PKI)) Y_{MAX}(PKAC) - PKAC]$$

$$\frac{dPP2A}{dt} = \frac{1}{\tau_{PP2A}}[W_{PP2A} Y_{MAX}(PP2A) - PP2A]$$

$$\frac{dIKs}{dt} = \frac{1}{\tau_{Iks}}[OR(f_{act}(PKAC), f_{inhib}(PP1)) Y_{MAX}(IKs) - IKs]$$

$$\frac{dICa}{dt} = \frac{1}{\tau_{ICa}}[OR(f_{act}(PKAC), OR(f_{inhib}(PP2A), f_{inhib}(PP1))) Y_{MAX}(ICa) - ICa]$$

$$\frac{dRyR}{dt} = \frac{1}{\tau_{RyR}}[OR(f_{act}(PKAC), OR(f_{inhib}(PP2A), f_{inhib}(PP1))) Y_{MAX}(RyR) - RyR]$$

$$\frac{dInhib1}{dt} = \frac{1}{\tau_{Inhib1}}[OR(f_{act}(PKAC), f_{inhib}(PP2A)) Y_{MAX}(Inhib1) - Inhib1]$$

$$\frac{dTnI}{dt} = \frac{1}{\tau_{TnI}}[OR(f_{act}(PKAC), f_{inhib}(PP2A)) Y_{MAX}(TnI) - TnI]$$

$$\frac{dPLB}{dt} = \frac{1}{\tau_{PLB}}[OR(f_{act}(PKAC), f_{inhib}(PP1)) Y_{MAX}(PLB) - PLB]$$

$$\frac{dPP1}{dt} = \frac{1}{\tau_{PP1}}[f_{inhib}(Inhib1) Y_{MAX}(PP1) - PP1]$$

*Note that f_{act} and f_{inhib} are the same activation and inhibition functions described for the previous section for the toy network. AND and OR functionality is also as described in the previous section.

****Parameter Estimates for Figure 7 and Figure S5 only****:

Listed below are the adjusted parameter values determined by the nonlinear least squares fitting procedure. These parameters were used to produce the normalized-Hill model traces shown in Figure 7 of the main text and Figure S5. In all other cases, default parameter values were used.

$\tau_{B1AR} = 0.1$
$\tau_{B1ARPG} = 0.611$
$\tau_{B1ARPA} = 287.97$
$\tau_{PLB} = 9.69$

For the remaining reaction parameters, the description implies that species "A" is acting on species "B". For example, if the weight for the activation reaction between GRK and B1ARPG were altered, this would be represented as W(GRK→B1ARPG). These parameters are present in the differentials for species B in such cases (e.g. dydt(B1ARPB) is altered in the example).

W(GRK→B1ARPG) = 0.537
W(PKAC→B1ARPA) = 0.95
W(PKAC→PLB) = 0.5947
EC50(AC→cAMP) = 0.5324
EC50(cAMP→PKAC) = 0.4881
EC50(PKAC→PLB) = 0.3128

Finally, an additional term 'W_b' was added to represent constitutive B1AR receptor activity. The differential equation for B1AR was altered to include an OR reaction between the basal rate and the original effectors of the receptor described above.

$W_b = 0.225$

$$\frac{dB1AR}{dt} = \frac{1}{\tau_{B1AR}}[OR(f_{act}(NE)f_{inhib}(B1ARPG)f_{inhib}(B1ARPA),W_b)Y_{MAX}(B1AR) - B1AR]$$
