# Supplementary figures and images for: Modeling cardiac β-adrenergic signaling with normalized-Hill differential equations: comparison with a biochemical model
Source: BMC Syst Biol. 2010 Nov 18;4:157. doi: 10.1186/1752-0509-4-157 (PMC2993667; doi:10.1186/1752-0509-4-157)

A

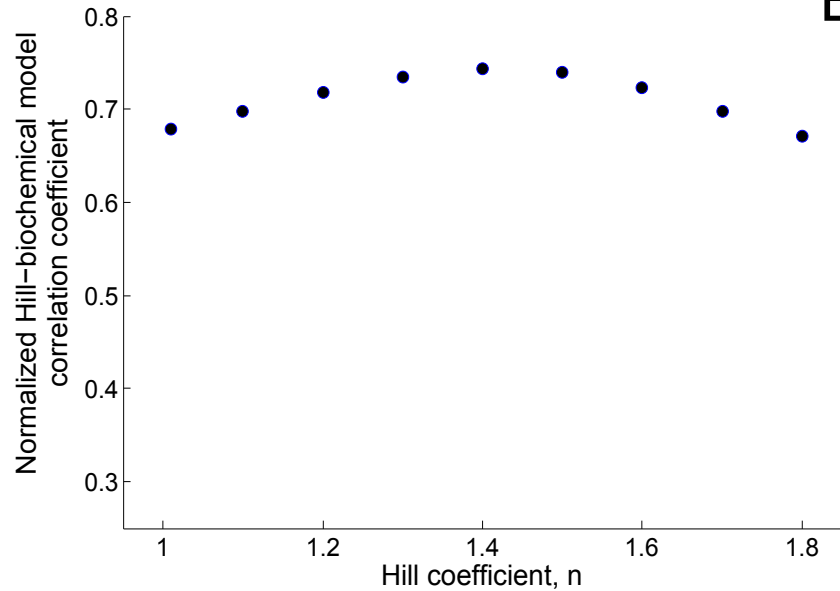

B

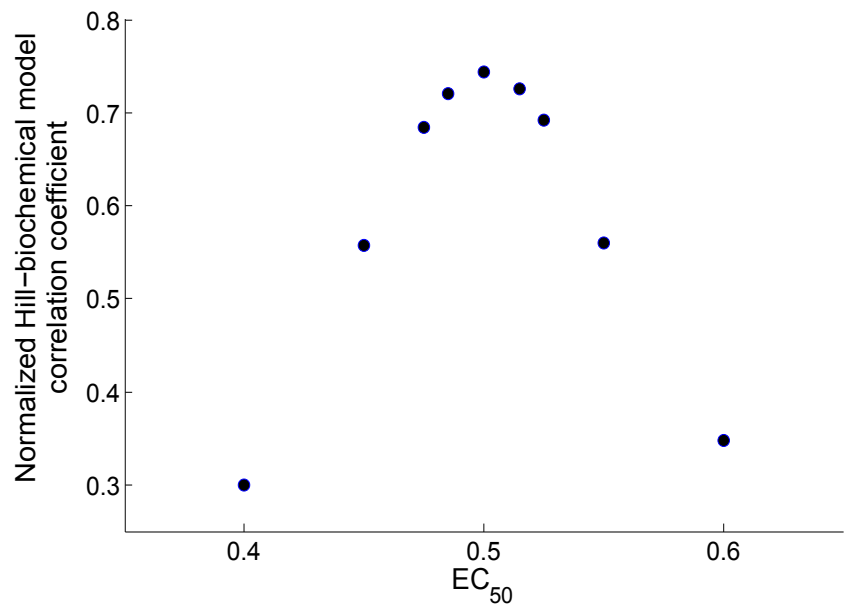

Supplement: Additional file 2 — Figure S1: Dependence of model accuracy on choices for default parameters. A) Varying the choice of default Hill coefficient has little effect on the predictive capability of the normalized-Hill model compared to the detailed biochemical model, though n = 1.4 is optimal. B) Strength of normalized-Hill predictions are very sensitive to the choice of default EC50, where model predictions dramatically worsened as default EC50 deviated from the intuitive value of 0.5. [file 1752-0509-4-157-S2.PDF]

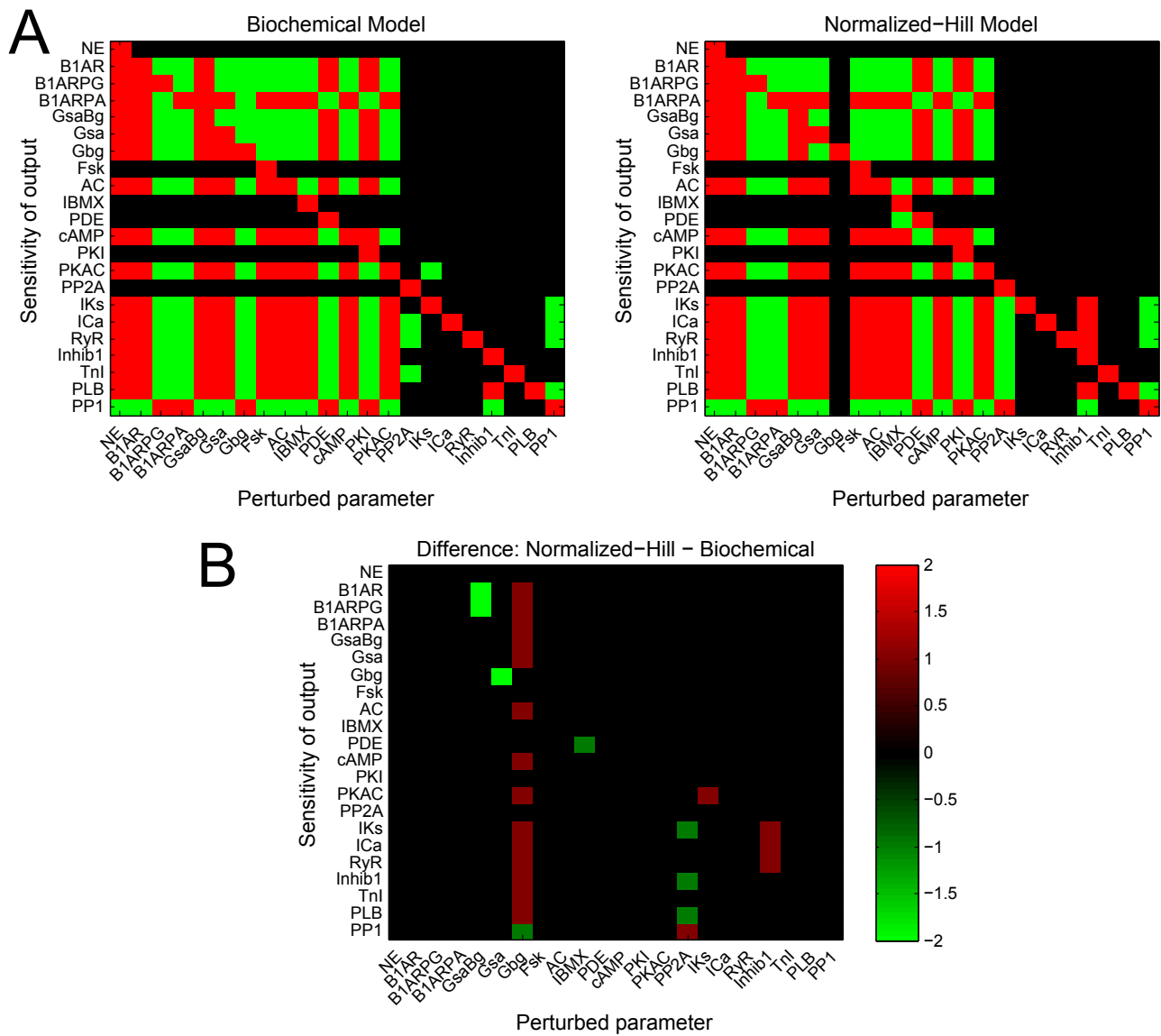

Figure S2

Supplement: Additional file 3 — Figure S2: Qualitative comparison of sensitivity matrices from normalized-Hill and biochemical β-adrenergic models. A) Individual elements of each sensitivity matrix were re-classified as "activating" (red), "inhibiting" (green) or "neutral" (black), in which a threshold sensitivity of +/- 0.003 was determined by visual inspection of a histogram of sensitivities for each model. B) Difference plot showing regions of qualitative discrepancy between normalized-Hill model and the biochemical model. Globally, the two models showed good agreement in terms of individual sensitivity types, with 457 out of 484 (94%) individual sensitivities qualitatively matching. Of the 27 mismatches, only 3 sensitivities were in opposite directions (difference of -2; 0.62% of the total, see main text for further details). [file 1752-0509-4-157-S3.PDF]

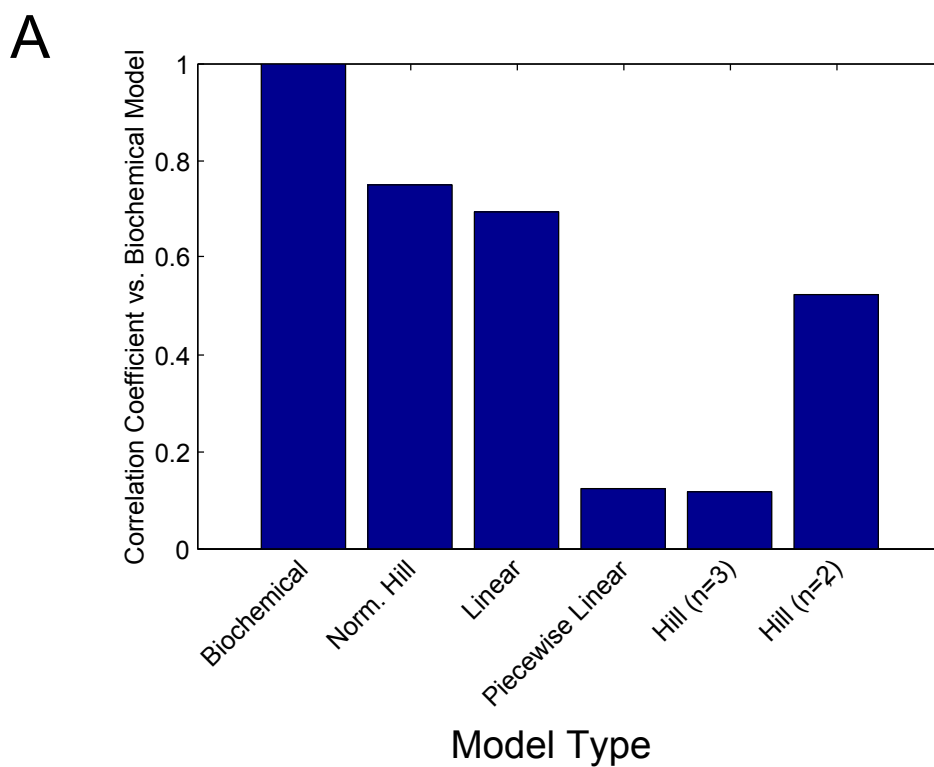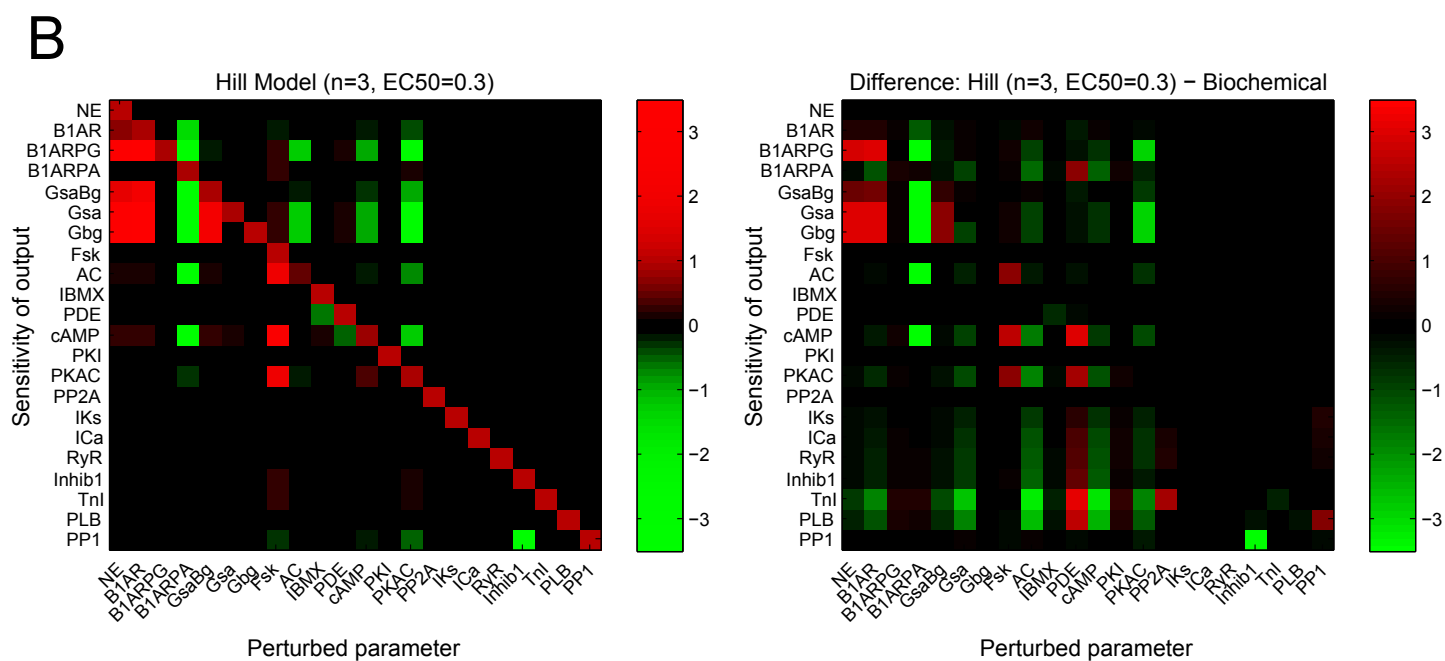

Figure S3

Supplement: Additional file 4 — Figure S3: Comparison of biochemical model with normalized-Hill model and alternative modeling implementations. Differential equation models of the same β-adrenergic network were generated using linear, piece-wise linear, or traditional Hill activation functions. All other parts of the model were kept constant, including time constants, reaction weights, AND/OR logic, and network topology. A) Pearson correlation coefficients were computed by comparing the sensitivity matrix of each model against the sensitivity matrix of the biochemical model. The normalized-Hill approach produces the highest level of agreement. B, left) Sensitivity matrix using Hill activation functions of n = 3 and EC50 = 0.3, as used previously for default parameters a for T-cell signaling network [17]. B, right) Difference between the sensitivity matrices from the traditional Hill model (n = 3, EC50 = 0.3) and the biochemical model, indicating areas of discrepancy. Sensitivity matrices for remaining modeling approaches are shown in Additional File 5, Figure S4. [file 1752-0509-4-157-S4.PDF]

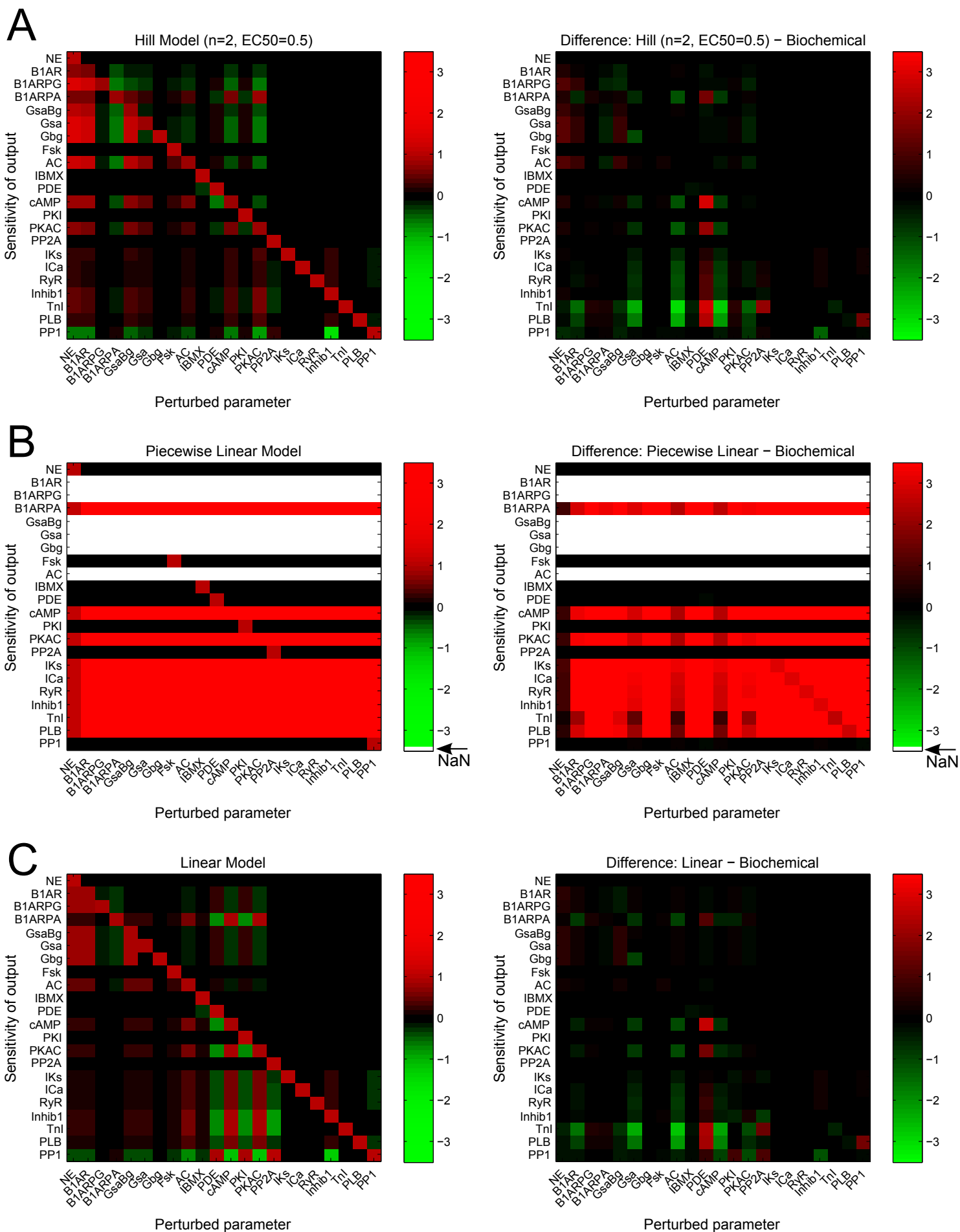

Figure S4

Supplement: Additional file 5 — Figure S4: Sensitivity matrices and differences from the biochemical model for β-adrenergic models implemented with "piece-wise linear", Hill, and linear activation functions. A) Traditional Hill activation with n = 2 and EC50 = 0.5. B) Activation was implemented as fact(x < 0.5) = 0, fact(x≥0.5) = 1 as described for "piece-wise linear differential equations" in [9,15]. White indicates values with no Real solution (NaN), which were excluded from computation of the correlation coefficient. The poor agreement is related to the limited number of possible steady-state values using this activation function. C) Linear activation functions, implemented as fact(x) = x. [file 1752-0509-4-157-S5.PDF]

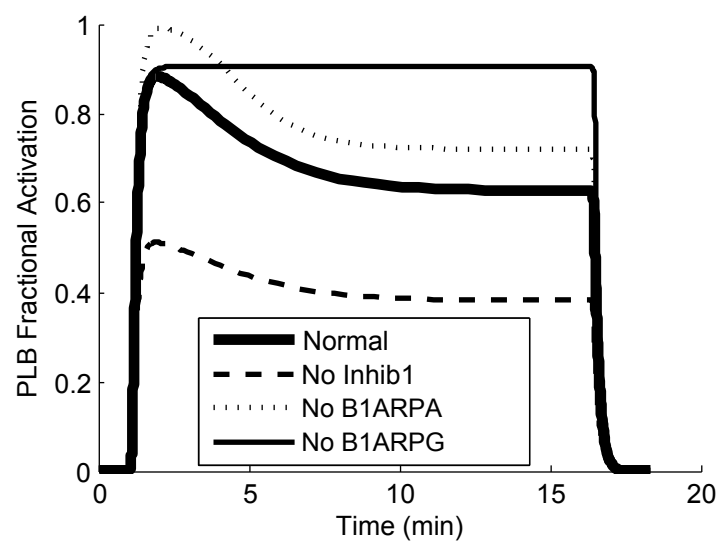

Figure S5

Supplement: Additional file 6 — Figure S5: Dynamic roles for feedback and feed-forward loops in the β-adrenergic network in the adjusted normalized-Hill Model. Using the adjusted model described in Figure 7, perturbations to feedback and feed-forward loops were performed as in Figure 5. The thick solid line is the response of the adjusted model to transient NE exposure, showing moderate PLB activation and partial adaptation to constant input. Also shown are simulations in which either the inhibitor-1 coherent positive feed-forward loop (thick dashed line; WPKAC-Inhib1 = 0), the GRK negative feedback loop (thin dashed line; WGRK-B1ARPG = 0) or the PKAC negative feedback loop (thin solid line; WPKAC-B1ARPA = 0) were eliminated. Inhibitor-1 amplified PLB activation without affecting PLB dynamics. Both GRK and PKAC feedback loops decreased PLB phosphorylation via β1-adrenergic receptor desensitization, though PKAC controlled steady-state adaptation while GRK feedback attenuated the PLB response. While the adjusted model does not exhibit the damped PLB oscillations of the default model (Figure 5), the roles of these feed-forward and feedback loops were largely maintained. [file 1752-0509-4-157-S6.PDF]

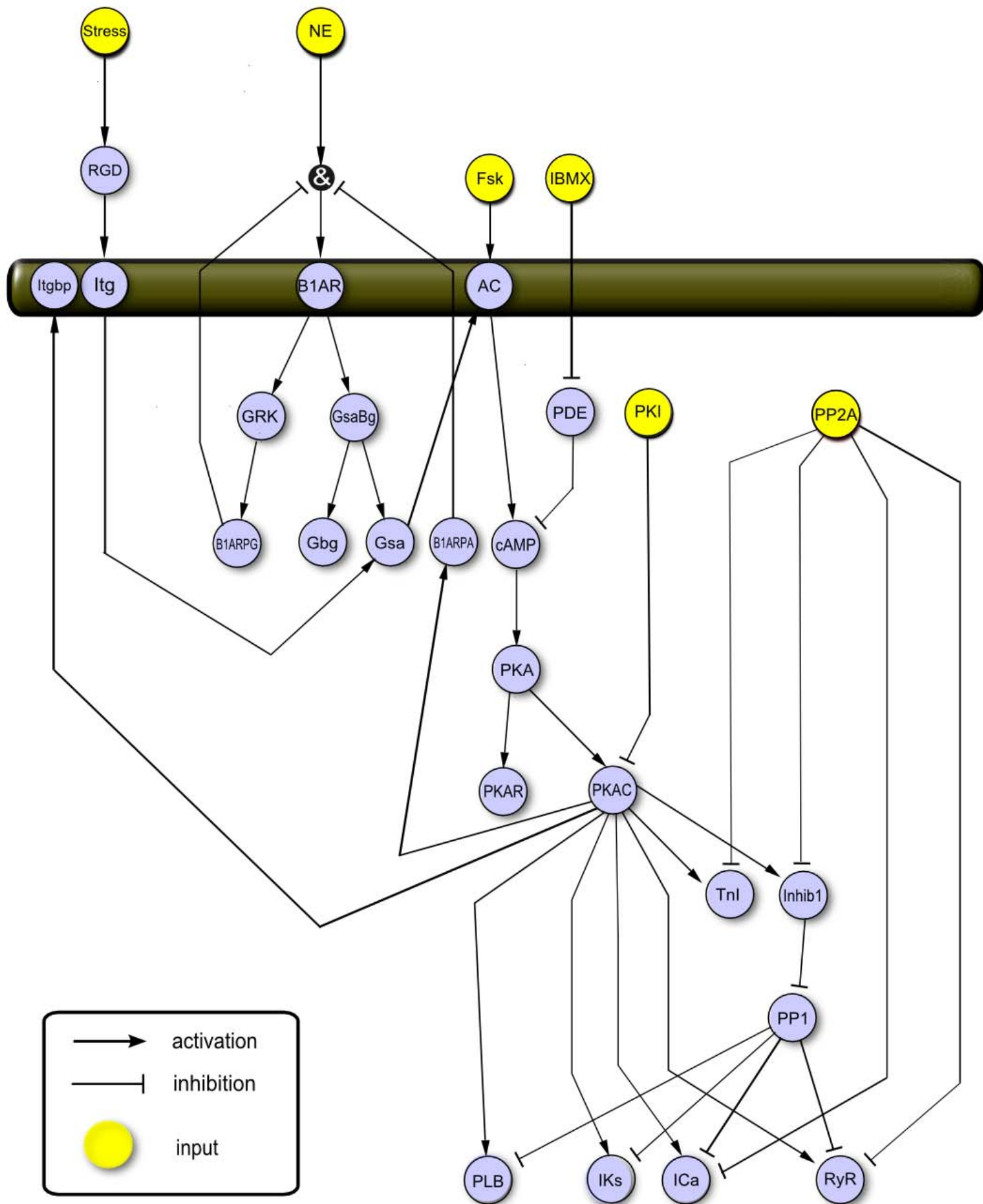

Supplement: Additional file 7 — Figure S6: Schematic of the β-adrenergic network with addition of integrin signaling. Based on recent protein interaction data http://www.pathwaycommons.org/pc/, reactions involving mechanical stress, RGD-coupled microbeads, integrins (Itg), and PKA-mediated integrin phosphorylation (Itgbp) were added to the model to demonstrate the extensibility of the normalized-Hill modeling approach. [file 1752-0509-4-157-S7.PDF]
